# Burden of diabetes attributable to dietary cadmium exposure in adolescents and adults in China

**DOI:** 10.1007/s11356-023-29424-6

**Published:** 2023-09-04

**Authors:** Shan Li, Muhadasi Tuerxunyiming, Zhe Sun, Su-yang Zheng, Qing-bai Liu, Qing Zhao

**Affiliations:** 1 Department of Preventive Medicine, Medical College, Shihezi University, Shihezi, 832002 China; 2 School of Medicine, Hangzhou City University, Hangzhou, 310015 Zhejiang Province China; 3 Department of Endocrinology, The First Hospital of Qiqihar, The Affiliated Qiqihar Hospital of Southern Medical University, Qiqihar, 161005 Heilongjiang China; 4 Department of Orthopaedics, Nanjing First Hospital, Nanjing Medical University, Nanjing, 210006 China; 5 Department of Endocrinology, Lianshui People’s Hospital of Kangda College Affiliated to Nanjing Medical University, Huai’an, 223400 Jiangsu Province China

**Keywords:** China, Cadmium, Diabetes mellitus, Adolescents and adult**s**, Attribution analysis, Foodborne disease burden, Disability adjusted life years

## Abstract

At present, the health risk assessment of cadmium exposure has become a major focus of environmental health research. However, there is still a lack of systematic research on the burden of diabetes (DM) attributable to dietary cadmium exposure in adolescents and adults in China. Using the top-down method, the blood cadmium level (B-Cd) of Chinese adolescents and adults from 2001 to 2023 was combined with the relative risk (RR) of cadmium-induced diabetes to calculate the population attribution score (PAF). Subsequently, PAF was used to assess the disease burden (DB) of diabetes caused by cadmium exposure, expressed in disability adjusted life years (DALYs), and attribution analysis was carried out for cadmium exposure from different sources. The average blood cadmium concentration in Chinese adolescents and adults was 1.54 ± 1.13 µg/L, and the burden of DM attributable to cadmium exposure was 56.52 (44.81, 70.33) × 10^5^ DALYs. The contribution rate of dietary cadmium exposure was 59.78%, and the burden of DM attributable to dietary cadmium exposure was 337.86 (267.85, 420.42) × 10^8^ DALYs. In addition, the highest blood cadmium concentrations were found in Henan, Shanxi, and Jiangxi provinces, while the highest burden of DM attributable to cadmium exposure was found in Jiangsu, Henan, and Guangdong provinces. Cadmium exposure is a risk factor for DM, and we need to take comprehensive action to reduce the burden of DM attributable to dietary cadmium from health, economic, and social perspectives.

**Supplementary Information:**

The online version contains supplementary material available at 10.1007/s11356-023-29424-6.

## Introduction

Cadmium is a toxic heavy metal that is difficult to degrade in the environment. In industrial production, cadmium is widely used in the production of batteries, plastic stabilizers, and coatings. The cadmium emitted during this process can cause serious pollution to soil, air, water, and food (Sun et al. 2017). Due to the high mobility of cadmium in the soil–plant system, it is easily absorbed by plants, causing more widespread harm through the food chain (Liu et al. 2022). Smoking is an important source of cadmium exposure for the general population because tobacco contains high levels of cadmium. However, for non-smokers and non-occupational populations, the main source of cadmium exposure is diet (He et al. 2013). The biological half-life of cadmium is 10–30 years, and the human body can hardly complete metabolism and elimination. In addition, scientific research has shown that cadmium has no beneficial effects on human health, so studying the health effects of low-level heavy metal exposure on occupational workers and the general population is of great significance (Wang et al. 2020).

Exposure to cadmium is closely related to metabolic diseases such as DM, obesity, and thyroid diseases. There is a dose–response relationship between cadmium exposure and DM, meaning that the higher the cadmium concentration, the higher the incidence of DM (Filippini et al. 2022). Epidemiological studies have shown that the increase in cadmium concentration is positively correlated with the incidence of DM (Satarug et al. 2017). Studies have also found that higher chronic cadmium exposure in adults may lead to an increase in fasting blood glucose, which in turn leads to the occurrence of DM (Xiao et al. 2021; Wang et al. 2022). Experimental studies have shown that cadmium exposure can disrupt lipid metabolism in pancreatic β-cells, induce pancreatic inflammation, and thus lead to the occurrence of DM (Hong et al. 2021). In addition, cadmium exposure disrupts glucose metabolism and exacerbates DM, with a greater risk of developing DM when exposed to cadmium (Li et al. 2022). The above studies indicate that cadmium exposure may be an important risk factor for the occurrence of DM, and effective prevention and control strategies are urgently needed to reduce the burden of DM attributable to cadmium exposure.

Currently, DM has become an increasingly serious global public health problem. Since 1990, the global burden of DM has significantly increased. The trends and levels of DM burden vary greatly among different regions and countries (Lin et al. 2022). It is estimated that there are 114 million DM patients in China, and the burden of DM attributable to this is also increasing, so it is particularly important to identify and develop sustainable intervention measures on time (Luo et al. 2020). It has been reported that diet is the main source of non-occupational cadmium exposure in the population, and cadmium intake through diet can pose potential hazards to human health in many regions (Zhang et al. 2018). However, there is currently no systematic study investigating the DM burden attributable to dietary cadmium exposure in China. Therefore, this study mainly explores the impact of dietary cadmium exposure on DM and quantifies the DB of DM attributable to dietary cadmium exposure.

The aim of this study is to explore the DM burden attributable to dietary cadmium exposure. Firstly, the B-Cd distribution of Chinese adolescents and adults of different genders and regions was estimated, and the PAF was calculated based on previous research methods (Liu et al. 2022). Then, the DM burden attributable to cadmium exposure was evaluated by multiplying the PAF with the DALY attributable to all reasons for DM in the Chinese population. Considering the different sources of cadmium exposure, we used exposure assessment models and exposure parameters of the Chinese population to calculate the contribution rate of each pathway, and finally attributed the DB attributable to DM to individual cadmium exposure sources. This study quantifies the DM burden attributable to cadmium exposure in China, which complements the existing relevant information and provides references for public health policies.

## Material and methods

### Overview

**Fig. 1:**
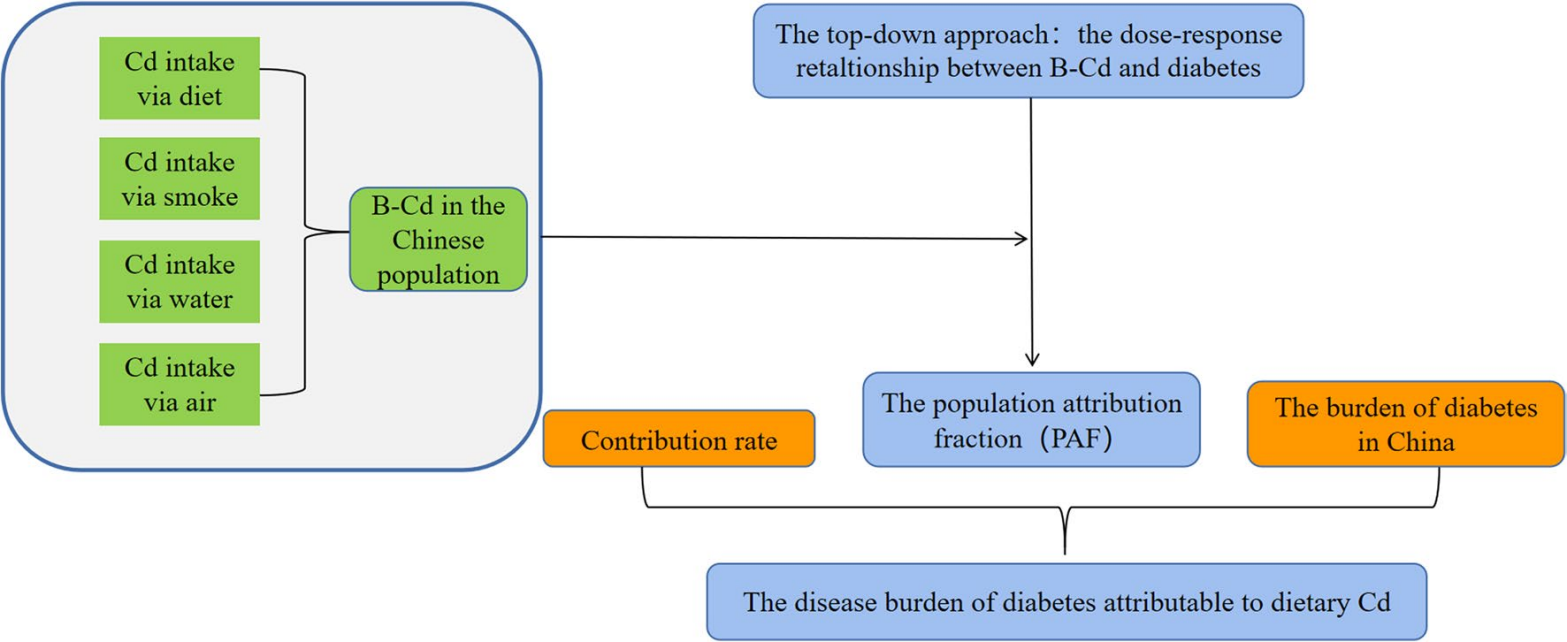
Framework diagram in the present study. OIM observed individual means (Yan et.al. 2022)

As shown in Fig. 1, firstly, the PAF was calculated by the distribution proportion of B-Cd in the population and the corresponding RR value induced by cadmium and then multiplied by the DM burden attributable to all reasons to determine the DB attributed to cadmium exposure. Secondly, the cadmium absorption and contribution rate from different sources were calculated, and finally, the DM burden attributed to cadmium exposure from different sources was estimated.

### B-Cd in adolescents and adults of China

#### Literature retrieval and data extraction

A reasonable search strategy was adopted to retrieve literature on blood cadmium concentrations in Chinese adolescents and adults reported between January 1, 2001, and April 1, 2023, from PubMed, Web of Science, China National Knowledge Infrastructure (CNKI), Wanfang Database, and China Biomedical Literature Database (CBMdisc). The retrieved articles were evaluated based on the following inclusion criteria: (1) the study subjects were Chinese residents aged ≥ 15 years; (2) the study subjects did not include pregnant women; (3) the sample collection time was after 2000; (4) blood samples were collected from veins or fingertips; (5) data collection was from non-cadmium-polluted areas; and (6) the results included the sample size, arithmetic mean, and standard deviation of cadmium concentration. Literature that did not meet one or more of the above criteria was excluded. Two independent reviewers screened the results to assess whether they met the inclusion criteria and whether any disagreements were resolved by a third party. In addition, the potential publication bias of the included literature was analyzed.

#### Pooling the B-Cd

Firstly, following the recommendation of Hozo et al. (2005) for non-arithmetic mean and standard deviation of raw data, the arithmetic mean and standard deviation of extreme values reported in the studies were included. Secondly, the arithmetic mean and standard deviation of blood cadmium concentrations in different genders and regions were combined using a weighted method. The formula for the combination is as follows:

1 $${\rm X}=\sum \overline{x }\cdot n/\sum n$$

2 $$S=\sqrt{\sum (({S}^{2}}+{x}^{2})\cdot n)/\sum n-{(\sum \overline{x }\cdot n/\sum n)}^{2}$$

*X* is the weighted average of the means of each sample, *S* is the weighted standard deviation of the standard deviations of each sample, *n* is the sample size, and $\overline{x }$ is the sample mean. The standardized unit for blood cadmium concentration is µg/L.

### Distribution proportion of B-Cd in the population

According to the calculation of the area under the logarithmic normal distribution curve in Microsoft Excel, the proportion of individuals exposed to cadmium is determined by grouping the blood cadmium levels of adolescents and adults into ranges of 1.5–2.0, 2.0–2.5, and ≥ 2.5 µg/L.

3 $$N=1-\mathrm{LOGNORMDIST}\left[\mathrm{x},\mathrm{ln}\left(X\right),\mathrm{ln}\left(\mathrm{S}\right)\right]$$

Here, *x* is the minimum value in each B-Cd group (5, 10, 15, and 20 µg/dL) and *X* and *S* are the AM and SD of each B-Cd group.

### Calculating DALYs attributable to cadmium exposure

Exposure to cadmium can lead to adverse health consequences such as DM, cardiovascular disease, and damage to the reproductive system. However, research on quantifying these consequences through DALY is limited. Disability-adjusted life years are a standard indicator used to evaluate the DB attributable to environmental exposures (Gao et al. 2015).

This study evaluated the burden of disease attributable to cadmium exposure in Chinese adolescents and adults, using DM as the endpoint indicator. By calculating the population distribution proportion of B-Cd and the corresponding DM RR value attributable to cadmium exposure, the PAF was calculated, and then the burden of DM attributable to cadmium exposure was calculated in combination with the total burden of DM.

#### The dose-response relationship and relative risk (RR) between B-Cd and DM mellitus

This study used data calculated from a previously published systematic review article (Filippini et al. 2022). The review followed standard literature search and meta-analysis processes and studied the dose–response relationship between B-Cd and DM. According to the results analyzed by Filippini et al. the relationship between B-Cd (µg/L) and DM is a significant linear positive correlation. Therefore, the concentration of B-Cd is divided into three levels based on the slope of the curve (1.5–2.0, 2.0–2.50, > 2.50 µg/L), and the corresponding RR (1.47, 2.43, 4.00) for each level is the continuous RR average value between regions.

#### Population attributable fraction (PAF)

The population attributable fraction (PAF) is a commonly used indicator in the burden of disease research, which indicates the proportion of disease cases that could be avoided if the risk exposure of a population was reduced to an ideal level (O'Connell and Ferguson 2022). To calculate the burden of DM attributed to cadmium exposure, PAF is calculated based on the population distribution of various blood cadmium levels and corresponding relative risks (RR) of cadmium-induced DM, and then combined with the overall burden of DM.

The PAF calculation formula is as follows:

4 $$PAF=\frac{{\sum }_{i=1}^{n}{P}_{i}(R{R}_{i}-1)}{{\sum }_{i=1}^{n}{P}_{i}(R{R}_{i}-1)+1}$$

Here, *P*_i_ is the proportion of population distribution in each B-Cd group i (1.5–2.0; 2.0–2.50; and > 2.50 µg/L) from Formula (3). *RR*_i_ is the relative risk of DB in each B-Cd group.

### Estimating the contribution of each source to lead exposure

Humans can be exposed to cadmium through diet, smoking, air, and water. We estimated the cadmium intake from different sources and calculated the corresponding contribution rates.

We used the model from (Boon et al. 2011) to assess cadmium exposure. The model parameters included consumption levels of cadmium from different sources and cadmium concentrations in the population. The data on cadmium concentrations in the population were obtained (Gu 2019; Luo and Xiang 2018; Filippini et al. 2022), while the data on cadmium consumption levels were obtained from the “Chinese Population Exposure Factors Handbook” (Zhao and Duan 2014). Based on the available parameters, we calculated the cadmium intake from smoking, air, water, and diet and calculated the corresponding contribution rates to elucidate the impact of cadmium exposure from each source.

5 $$\mathrm{Contribution\ rate }\left(\%\right)=\mathrm{Cd\ uptake\ per\ source}/\mathrm{Cd\ uptake\ from\ all\ sources}$$

### Estimating the DALYs attributable to each source of cadmium exposure

According to Formula (5), the cadmium intake and contribution rate through four exposure pathways are obtained. Then, the cadmium contribution rate of the four exposure pathways is multiplied by the DM burden attributable to cadmium exposure. This gives the DM burden attributed to cadmium exposure from different sources.

6 $$\mathrm{Attributable\ DALYs}=\mathrm{DALYs\ of\ cadmium}-\mathrm{induced\ DM}\times \mathrm{contribution\ rate}$$

### Statistical analysis

Statistical analysis was performed using STATA 12.0 (STATA Corp, College Station, TX, USA). Funnel plots, Begg’s correlation (Begg and Mazumdar 1994), and Egger’s linear regression (Egger et al. 1997) tests were used to assess potential publication bias. All tests were two-tailed, and *P* > 0.05 indicates statistical significance.

## Results

### B-Cd of adolescents and adults in China

This study retrieved a total of 1276 studies from five databases. After removing duplicate or excluded publications, 46 studies were finally included, covering 19 provinces, autonomous regions, and municipalities nationwide (Table S1, Figure S1). After analyzing the included studies, we did not find any potential publication bias (Figure S2).

**Fig. 2:**
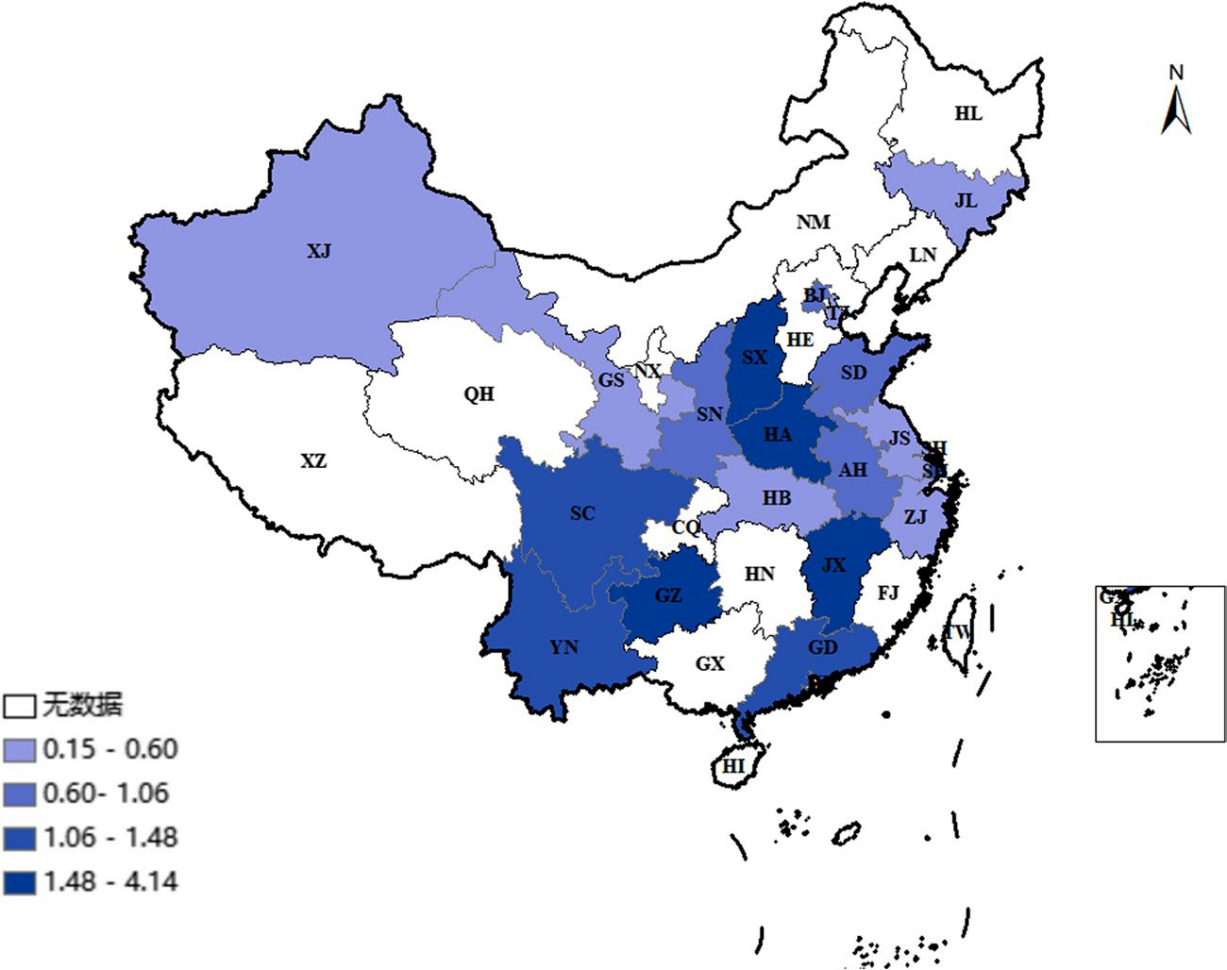
The distribution of Chinese adolescents and adults B-Cd, 2001–2023

This study evaluated the burden of DM resulting from cadmium exposure in Chinese adolescents and adults. It included 56,191 individuals from 19 provinces, autonomous regions, and municipalities in China. The sample consisted of 12,721 males and 15,130 females, with average blood cadmium concentrations of 1.54 ± 1.13, 1.81 ± 1.22, and 1.64 ± 0.97 µg/L, respectively. The study also compiled the blood cadmium levels of Chinese adolescents and adults by region. The highest blood cadmium concentrations were found in Henan, Shanxi, and Jiangxi (4.14, 2.84, and 2.82 µg/L, respectively), while the lowest were found in Gansu, Xinjiang, and Tianjin (0.17, 0.39, and 0.60 µg/L, respectively) (Table 1, Fig. 2).

**Table 1:** B-Cd levels of the adolescents and adults in China, 2001–2023

Groups	Sample size	AM (µg/L)	ASD (µg/L)
Gender			
Males	12,721	1.81	1.22
Females	15,130	1.64	0.97
Both	56,191	1.54	1.13
Regions			
Beijing	355	0.95	0.38
Tianjin	25	0.60	0.13
Gansu	293	0.17	0.12
Shanxi	503	2.82	1.95
Shaanxi	477	1.08	1.17
Jilin	671	0.84	0.70
Shandong	241	1.17	0.96
Henan	402	4.14	2.54
Xinjiang	50	0.39	0.86
Shanghai	2645	1.49	1.70
Jiangsu	3473	0.62	1.82
Zhejiang	3473	0.79	0.85
Anhui	480	1.06	0.26
Guangdong	6384	1.33	0.64
Hubei	189	0.83	0.34
Jiangxi	1826	2.84	0.35
Sichuan	30	1.39	0.12
Guizhou	402	2.50	1.07
Yunnan	356	1.35	1.21

*B-Cd blood cadmium level, ^&^*AM* arithmetic mean, ^#^*ASD* arithmetic standard deviation

### DB of DM attributable to cadmium exposure in Chinese adolescents and adults

The PAFs were calculated for different sexes and regions based on the population distribution ratio and *RR*_i_ value to evaluate the burden of DM attributable to cadmium exposure in this study. The results showed that the PAF for males was higher than that for females, and the PAF values were higher in some regions such as Jiangxi, Henan, and Guizhou than in other regions (Table S2, S3).

After combining the PAF results, the estimated DALYs for DM attributable to cadmium exposure in Chinese adolescents and adults in 2017 were 30.89 (24.00, 38.64) × 10^5^ DALYs for males and 27.01 (21.01, 33.87) × 10^5^ DALYs for females. In terms of regional distribution, Guangdong, Henan, and Sichuan had the highest DALY values (4.72, 4.57, and 3.96 × 10^5^ DALYs, respectively), while Tianjin, Gansu, and Xinjiang had the lowest DALY values (0.39, 0.52, and 0.93 × 10^5^ DALYs, respectively) (Table 2).

**Table 2:** DALYs of DM attributed to Cd exposure in China, 2017

Index	Group	Population^*^	DALYs (× 10^5^)	DALYs Rate (/10^7^)
Gender^**#**1^	Males	571070388.3	30.89 (24.00, 38.64)	224.70 (174.55.27, 281.09)
	Females	585353155.3	27.01 (21.01, 33.87)	194.19 (151.04, 243.47)
	Both	1156422330	56.52 (44.81, 70.33)	204.34 (162.00, 254.28)
Regions^**#**2^	Beijing	19246359.22	0.98 (0.71, 1.31)	4.05 (2.97, 5.42)
	Tianjin	13756067.96	0.39 (0.29, 0.50)	2.53 (1.91, 3.28)
	Gansu	21734223.3	0.52 (0.41, 0.67)	1.97 (1.55, 2.52)
	Shanxi	31290048.54	1.87 (1.37, 2.47)	4.83 (3.54, 6.39)
	Shaanxi	32404126.21	0.97 (0.75, 1.24)	2.45 (1.88, 3.14)
	Jilin	23827669.9	1.29 (0.97, 1.67)	4.63 (3.48, 5.99)
	Shandong	82419902.91	3.91 (2.96, 5.08)	3.94 (2.98, 5.12)
	Henan	75884708.74	4.57 (3.60, 5.75)	4.75 (3.73, 5.97)
	Xinjiang	18915048.54	0.93 (0.72, 1.17)	3.82 (2.96, 4.81)
	Shanghai	21667475.73	1.32 (1.00, 1.71)	4.73 (3.58, 6.13)
	Jiangsu	69430825.24	3.32 (2.55, 4.24)	4.07 (3.13, 5.19)
	Zhejiang	49640776.7	1.96 (1.47, 2.55)	3.20 (2.39, 4.16)
	Anhui	50717233.01	2.45 (1.90, 3.12)	4.01 (3.10, 5.09)
	Guangdong	92779126.21	4.72 (3.48, 6.22)	4.04 (2.79, 5.32)
	Hubei	49736650.49	1.72 (1.35, 2.20)	3.11 (2.44, 3.98)
	Jiangxi	36308252.43	1.92 (1.47, 2.44)	4.03 (3.08, 5.12)
	Sichuan	70083737.86	3.96 (3.11, 4.97)	4.75 (3.72, 5.95)
	Guizhou	28285194.17	1.51 (1.18, 1.90)	4.46 (3.48, 5.62)
	Yunnan	39066747.57	1.88 (1.47, 2.35)	3.81 (2.99, 4.77)

^*&*^*DALYs* disability adjusted life years

*Data came from China Statistical Yearbook 2018

^#1^Data came from GBD 2019, ^#2^data came from (Zhou et al. 2019)

### Cadmium uptake and contribution rate of adolescents and adults in China

**Fig. 3:**
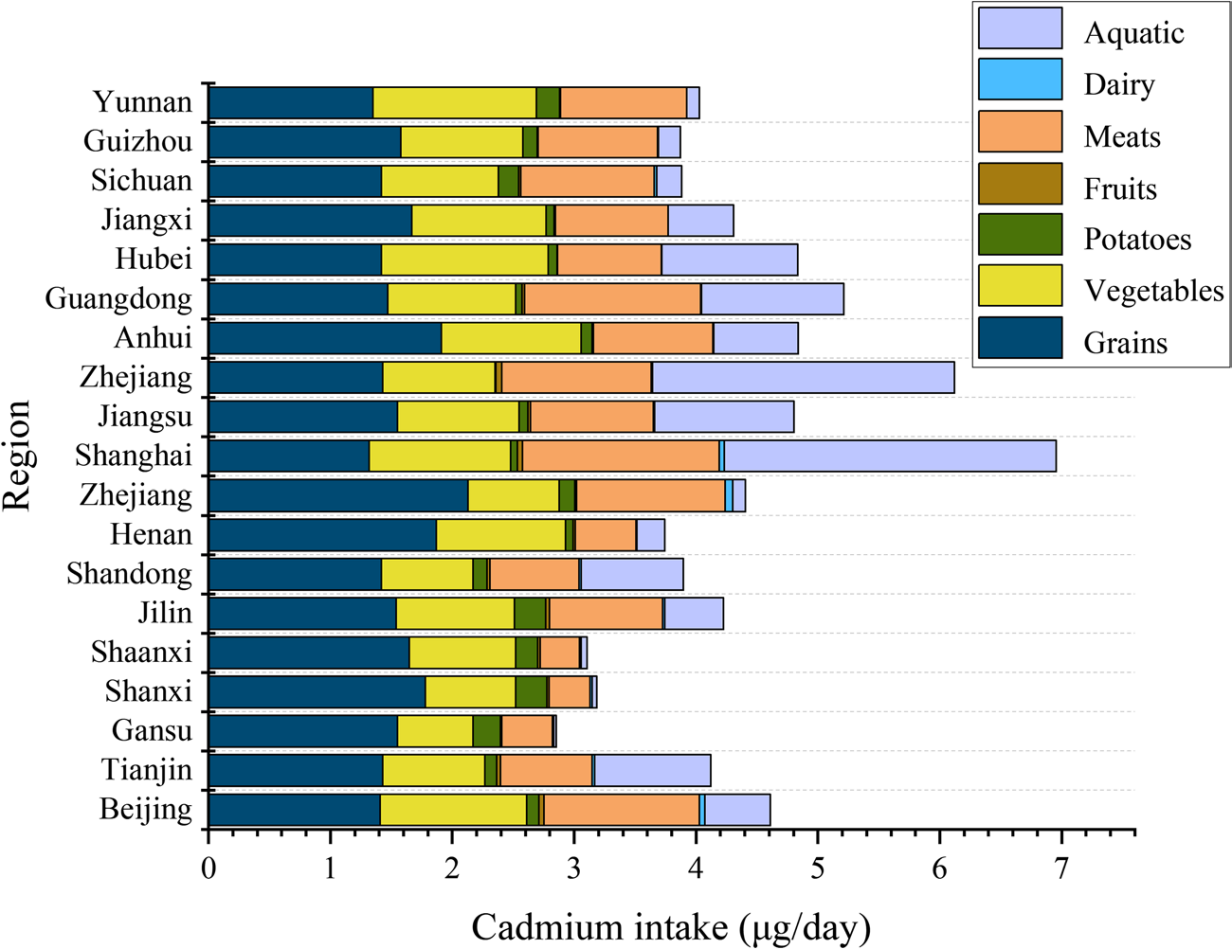
The cadmium intake of Chinese adolescents and adults B-Cd, 2001–2023

Cadmium can accumulate in the human body through various pathways and impact the occurrence of diseases. Therefore, this study utilized cadmium exposure parameters to evaluate the cadmium intake through different pathways in Chinese adolescents and adults. Our results indicate that the cadmium intake in Chinese adolescents and adults is approximately 7.73 µg/day, with significant differences in dietary cadmium intake among provinces. Furthermore, the average daily dietary intake from different sources is 4.62 µg/day (4.47 µg/day for males and 3.80 µg/day for females). Diet contributes the most to the total cadmium intake, accounting for 59.78%, followed by smoking, water, and air, which account for 37.84%, 1.91%, and 0.47%, respectively (Tables 3 and 4 and Figs. 2 and 3).

**Table 3:** Uptake of Cd exposure from various sources in China (µg/day)

	Smoking	Air	Water	Diet	Grains	Vegetable	Potatoes	Fruits	Meats	Dairy	Aquatic	Total
Total	2.93	0.04	0.15	4.62	1.55	1.02	0.13	0.02	1.10	0.02	0.79	7.73
Sex												
Males	5.42	0.04	0.23	4.47	1.66	1.07	0.12	0.01	0.95	0.01	0.64	10.16
Females	0.31	0.03	0.19	3.80	1.44	0.99	0.11	0.01	0.71	0.01	0.54	4.33
Regions												
Beijing	3.15	0.04	0.10	4.61	1.41	1.20	0.10	0.04	1.27	0.05	0.54	7.90
Tianjin	3.07	0.04	0.11	4.11	1.43	0.84	0.10	0.03	0.75	0.02	0.95	7.33
Gansu	2.77	0.02	0.20	2.85	1.55	0.62	0.23	0.01	0.42	0.01	0.02	5.85
Shanxi	3.10	0.04	0.25	3.18	1.78	0.74	0.26	0.02	0.34	0.01	0.04	6.58
Shaanxi	2.98	0.04	0.15	3.11	1.65	0.87	0.18	0.02	0.32	0.01	0.05	6.27
Jilin	3.12	0.02	0.06	4.22	1.54	0.97	0.26	0.03	0.93	0.02	0.48	7.42
Shandong	2.61	0.04	0.12	3.90	1.42	0.75	0.12	0.02	0.73	0.01	0.84	6.67
Henan	2.63	0.04	0.27	3.74	1.87	1.06	0.06	0.02	0.50	0.01	0.23	6.67
Xinjiang	1.76	0.04	0.24	4.41	2.13	0.75	0.13	0.01	1.22	0.06	0.10	6.44
Shanghai	2.58	0.04	0.07	6.96	1.32	1.16	0.05	0.04	1.61	0.04	2.72	9.64
Jiangsu	2.66	0.04	0.15	4.81	1.55	1.00	0.07	0.02	1.01	0.01	1.14	7.65
Zhejiang	2.87	0.04	0.09	6.12	1.43	0.92	0.01	0.05	1.23	0.01	2.47	9.13
Anhui	2.72	0.04	0.24	4.84	1.91	1.15	0.09	0.01	0.98	0.01	0.69	7.84
Guangdong	2.75	0.03	0.09	5.21	1.47	1.05	0.05	0.02	1.44	0.01	1.17	8.08
Hubei	2.98	0.04	0.08	4.84	1.42	1.37	0.07	0.01	0.85	0.00	1.11	7.94
Jiangxi	2.88	0.04	0.07	4.31	1.67	1.10	0.07	0.01	0.93	0.00	0.54	7.30
Sichuan	2.74	0.04	0.14	3.88	1.42	0.96	0.17	0.02	1.10	0.02	0.21	6.80
Guizhou	3.84	0.04	0.07	3.88	1.58	1.00	0.12	0.01	0.98	0.00	0.18	7.82
Yunnan	3.38	0.04	0.09	4.03	1.35	1.34	0.19	0.01	1.04	0.00	0.10	7.54

*The bioavailability of cadmium through diet, smoking, respiration, and drinking water was 10%, 70%, 25%, and 50%, respectively (MEP 2013)

**Table 4:** Contribution rates of cadmium exposure from various sources in China (%)

	Smoking	Air	Water	Diet
Total	37.84	0.47	1.91	59.78
Gender				
Male	53.30	0.40	2.28	44.02
Female	7.14	0.75	4.27	87.84
Regions				
Beijing	39.88	0.48	1.25	58.40
Tianjin	41.83	0.51	1.54	56.11
Gansu	47.32	0.37	3.48	48.83
Shanxi	47.15	0.57	3.86	48.42
Shaanxi	47.47	0.56	2.33	49.63
Jilin	42.03	0.32	0.80	56.86
Shandong	39.07	0.56	1.87	58.50
Henan	39.42	0.55	3.97	56.06
Xinjiang	27.30	0.57	3.74	68.38
Shanghai	26.75	0.38	0.75	72.12
Jiangsu	34.70	0.47	2.02	62.81
Zhejiang	31.48	0.39	1.03	67.09
Anhui	34.71	0.46	3.09	61.74
Guangdong	34.03	0.43	1.12	64.42
Hubei	37.50	0.46	1.06	60.99
Jiangxi	39.49	0.48	1.01	59.02
Sichuan	40.34	0.52	2.04	57.10
Guizhou	49.11	0.45	0.86	49.58
Yunnan	44.87	0.47	1.22	53.44

### DB of DM attributable to dietary cadmium exposure in adolescents and adults in China

We estimated the DB of DM attributed to dietary cadmium exposure based on the contribution of each exposure pathway. The burden of DM attributed to dietary cadmium exposure was estimated to be 337.86 (267.85, 420.42) × 10^6^ DALYs, with a burden in males and females of 135.99 (105.64, 170.11) × 10^6^ DALYs and 237.27 (184.54, 297.49) × 10^6^ DALYs, respectively. The burden of DM was higher in females than in males (Table 5).

**Table 5:** DALYs of DM attributed to Cd exposure from dietary sources in China, 2017

Regions/gender	Contribution rate of dietary Cd (%)	DALYs (× 10^6^)	DALYs rate (/10^8^)
Total	59.78	337.86 (267.85, 420.42)	1221.53 (968.43, 1520.06)
Gender			
Male	44.02	135.99 (105.64, 170.11)	989.13 (768.37, 1237.37)
Female	87.84	237.27 (184.54, 297.49)	1705.72 (1326.67, 2138.58)
Regions			
Beijing	58.40	5.71 (4.17, 7.63)	23.65 (17.33, 31.66)
Tianjin	56.11	2.17 (1.63, 2.80)	14.17 (10.69, 18.41)
Gansu	48.83	2.55 (2.01, 3.28)	9.64 (7.56, 12.31)
Shanxi	48.42	9.05 (6.61, 11.95)	23.38 (17.15, 30.95)
Shaanxi	49.63	4.81 (3.70, 6.15)	12.17 (9.34, 15.57)
Jilin	56.86	7.35 (5.51, 9.50)	26.32 (19.78, 34.08)
Shandong	58.50	22.90 (17.30, 29.73)	23.03 (17.41, 29.93)
Henan	56.06	25.64 (20.16, 32.25)	26.63 (20.92, 33.47)
Xinjiang	68.38	6.36 (4.92, 8.00)	26.11 (20.21, 32.87)
Shanghai	72.12	9.51 (7.21, 12.35)	34.08 (25.79, 44.19)
Jiangsu	62.81	20.87 (16.05, 26.62)	25.54 (19.63, 32.61)
Zhejiang	67.09	13.15 (9.84, 17.08)	21.48 (16.07, 27.89)
Anhui	61.74	15.14 (11.71, 19.23)	24.76 (19.13, 31.44)
Guangdong	64.42	30.41 (22.41, 40.07)	26.05 (17.98, 34.29)
Hubei	60.99	10.51 (8.24, 13.42)	18.99 (14.89, 24.28)
Jiangxi	59.02	11.30 (8.65, 14.39)	23.80 (18.19, 30.23)
Sichuan	57.10	22.63 (17.75, 28.37)	27.12 (21.24, 33.99)
Guizhou	49.58	7.49 (5.85, 9.44)	22.14 (17.28, 27.86)
Yunnan	53.44	10.03 (7.85, 12.55)	20.37 (15.97, 25.50)

## Discussion

This study primarily analyzed the burden of cadmium-induced DM mellitus in Chinese adolescents and adults. Based on the cadmium exposure levels of Chinese adolescents and adults, the study also estimated the exposure to blood cadmium in different regions of the country as well as the exposure levels of cadmium from different sources. The average blood cadmium exposure level for Chinese adolescents and adults in this study was 1.54 ± 1.13 µg/L, resulting in a burden of DM of 56.52 (44.81, 70.33) × 10^5^ DALYs. Furthermore, the study estimated the contribution of different sources of cadmium exposure and found that dietary cadmium accounted for as much as 59.78%, resulting in a burden of DM of 337.86 (267.85, 420.42) × 10^8^ DALYs.

Over the past 30 years, with the rapid development of urbanization and industrialization in China, many environmental issues including heavy metal pollution have arisen. As a result, the Chinese population has experienced relatively high levels of cadmium exposure (Wang et al. 2022). A cross-sectional survey report based on Chinese populations showed that the B-Cd levels for adult males and females in eastern China were 1.97 µg/L and 1.59 µg/L, respectively (Nie et al. 2016), which were higher than the B-Cd levels reported in this study for males (1.81 µg/L) and females (1.64 µg/L). This may be because this study covered a broader average blood cadmium level for different regions of China. In addition, this study also reported that the blood cadmium levels for Chinese adolescents and adults were 1.54 µg/L, which was significantly higher than the reported levels in developed countries such as Italy (0.53 µg/L) (Bocca et al. 2011) and Germany (0.38 µg/L) (Heitland and Köster 2006). This may be because blood cadmium varies by region, age, and race, and participants in low economic status and urban areas have significantly higher blood cadmium levels. Currently, the World Health Organization sets the blood cadmium level for the population at 10 µg/L, and China has also established relevant standards to limit cadmium intake (He et al 2013). However, some areas of China are still at high levels of cadmium exposure. Therefore, effective strategies are urgently needed to reduce the body burden of cadmium and prevent the public from long-term harmful effects.

Research has shown that alcohol consumption, smoking, lack of exercise, and unhealthy dietary habits are risk factors for DM, but there is also evidence that heavy metals, such as cadmium, are related to the development of DM (Ng et al. 2020; Li et al. 2019). Currently, widespread environmental cadmium exposure and its impact on DM is receiving increasing attention, partly due to the high incidence of DM and the role that cadmium exposure plays in driving this trend (Xiao et al. 2021). The distribution of B-Cd levels in DM due to environmental factors can be assessed using various methods. In this study, we used a top-down approach, whereby the PAF was calculated using population distribution ratios and corresponding RR values, and then multiplied by the total DM DALYs. The PAF for males and females in our study was 65.32% and 64.97%, respectively, indicating that eliminating cadmium exposure could reduce the incidence of DM by approximately 65.32 to 64.97%. Additionally, the burden of DM attributable to cadmium exposure was estimated to be 56.52 (44.81, 70.33) × 10^5^ DALYs, highlighting the high DM burden among Chinese adolescents and adults due to cadmium exposure, which calls for control measures.

Cadmium, a highly toxic heavy metal, can be absorbed by the human body through contaminated food, water, air, and even more through bioaccumulation (Wang et al. 2015). This study used the observed individual mean model to estimate the cadmium intake from each source, and the corresponding contribution rates were calculated. The study showed that the dietary pathway was the largest contributor to cadmium exposure (59.78%), consistent with previous research results (Zhong et al. 2015; Jean et al. 2018). In addition, the DM burden attributable to dietary cadmium was 337.86 (267.85, 420.42) × 10^6^ DALYs, significantly higher than other cadmium exposure pathways. Currently, dietary cadmium may be the main source of cadmium exposure for Chinese adolescents and adults, and food is mainly polluted by natural and human activities, such as industrial activities, transportation, and cadmium in agricultural practices, which can be absorbed by crops and aquatic plants (such as vegetables, rice, and seaweed) and by poultry and aquatic animals and accumulated in the gastrointestinal system. Therefore, the level of cadmium concentration in crops, animal offal, and aquatic plants (such as fish, shellfish, and seaweed) is proportional to the cadmium content in the environment (Zhang et al. 2018). To a lesser extent, cadmium can also reach high concentrations in drinking water (Peng et al. 2015). Therefore, controlling the cadmium concentration in these specific foods can reduce cadmium exposure to a certain extent, thereby reducing the incidence of DM.

This study evaluated the burden of DM attributable to cadmium exposure, but there are still some limitations. First, most of the studies included were cross-sectional, and it cannot be ruled out that the observed positive correlation between cadmium exposure and DM risk reflects the possibility of the effect of DM treatment or the disease itself on blood cadmium levels. Second, extracting and merging B-Cd data from the literature may have some errors. In addition, the RR used to calculate PAF is not specific to the Chinese population, so further research is needed to explore the dose–response relationship between cadmium and DM in the Chinese population. Finally, as there are no DALYs data specific to gender, this study calculated the total DALYs value based on the male-to-female ratio in the Chinese population and did not consider other indicators such as incidence and mortality rates in calculating DALYs. Numerous studies have shown that cadmium exposure is a driving force behind DM.

## Conclusion

In summary, our study indicates that exposure to cadmium can have serious impacts on public health, with dietary exposure to cadmium potentially contributing to the burden of DM. Our research quantified the burden of DM associated with dietary cadmium exposure, providing scientific evidence for the development of public health policies and offering a foundation for research aimed at preventing and reducing the burden of DM.

### Supplementary Information

Below is the link to the electronic supplementary material.

Supplementary file1 (DOCX 1.45 MB)

## Data Availability

The datasets used and/or analyzed during the current study are available from the corresponding author on reasonable request.
