# Supplementary material for: Burden of diabetes attributable to dietary cadmium exposure in adolescents and adults in China
Source: Environ Sci Pollut Res Int. 2023 Sep 4;30(46):102353–62. doi: 10.1007/s11356-023-29424-6 (PMC10567932; doi:10.1007/s11356-023-29424-6)
Supplement: Supplementary file 1 — Supplementary file1 (DOCX 1.45 MB) [file 11356_2023_29424_MOESM1_ESM.docx]

**Supplementary Material**

**Burden of diabetes attributable to dietary cadmium exposure in** **adolescents and adults in China**

**Part 1 Retrieval strategy and results**

**Table S1.** Retrieval strategy and results of B-Cd concentration in Chinese adolescents and adults

|  | Retrieval strategy | Results |
| --- | --- | --- |
| CNKI (In Chinese) | (Keyword:(blood cadmium))OR(Abstract: (blood cadmium)) Date: 2001/01/01-2023/04/01 | 392 |
| WAN FANG (In Chinese) | (Total:(blood cadmium)AND Total:(adolescents))OR (Total:(blood cadmium)AND Total:(adults))  Date: 2001/01/01-2023/04/01 | 47 |
| Web of Science | (blood cadmium OR blood cadmium levels OR BCLs OR BCd OR B-Cd OR CdB OR Cd-B OR  serum cadmium) AND (adults OR teenagers OR youngsters OR juvenile OR adolescents) AND (China OR Chinese)  Date: 2001/01/01-2023/04/01 | 454 |
| PubMed | blood **cadmium [tiab] OR** blood **cadmium level [tiab] OR (**BCLs **[tiab] OR Cd [tiab] OR BCd [tiab] OR B-Cd [tiab] CdB [tiab] Cd-B [tiab] OR serum cadmium [tiab] ) AND (adults [tiab] OR teenagers [tiab] OR youngsters[tiab] OR juvenile [tiab] OR adolescents [tiab] ) AND (China [tiab] OR Chinese [tiab] )** AND ("2001/01/01"[PDAT] : "2023/04/01"[PDAT]) | 380 |
| CBM disc  (In Chinese) | (“blood cadmium” [Full Field] AND “adolescent” [Full Field]) OR (“blood cadmium” [Full Field] AND “adult” [Full Field]) Date: 2001/01/01-2023/04/01 | 3 |

*CNKI=China National Knowledge Internet, ^&^CBM disc=China Biology Medicine Disc, and ^#^Wan Fang, of them, the professional academic database of China.

**Part 2 Flow chart for literature**


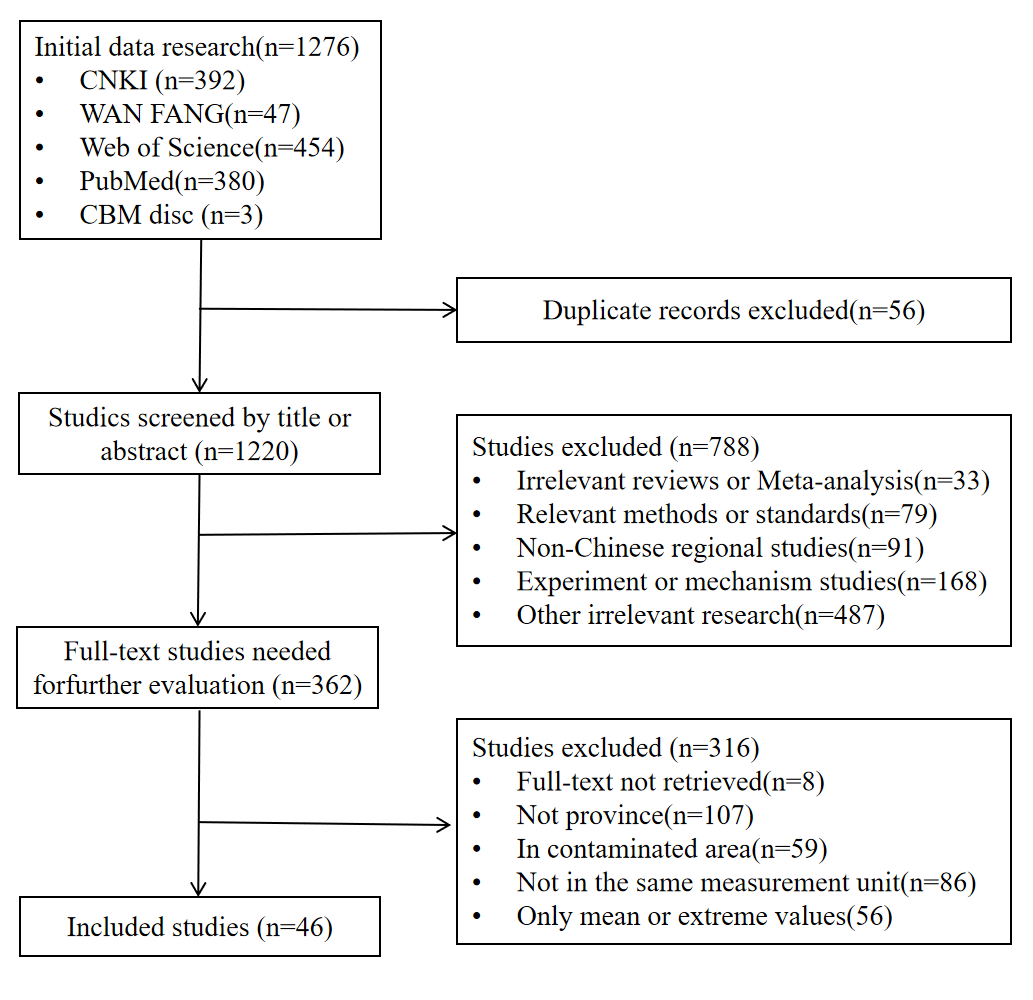


**Fig S1.**  Flow chart for literature search in Chinese and English databases

**Part 3 Publication bias analysis**


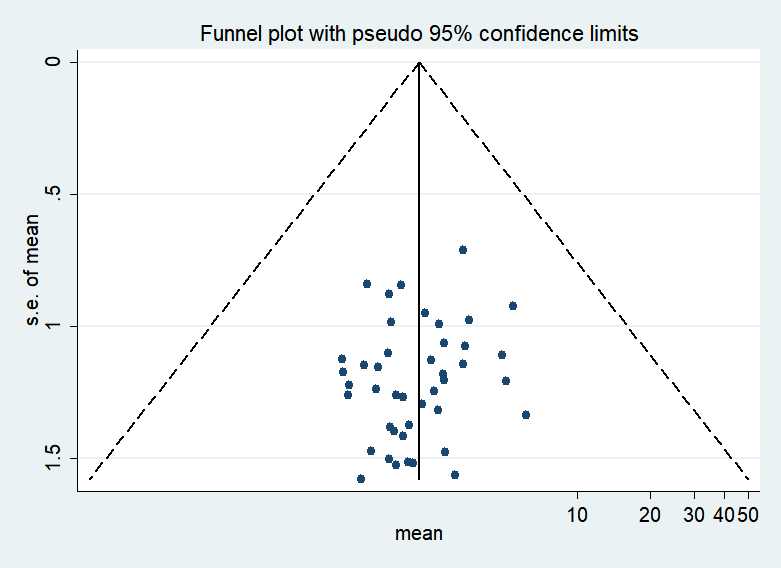


**Fig S2.** Funnel plot for potential publication bias analysis

Egger，*P*=0.264>0.05；Begg，z=0.47，*P*>0.642）

**Part 4 Distribution of the population with each B-Cd and health effects**

| **Table S2.** Population distribution proportion and health effects in different B-Cd intervals | | | | | | | | | |
| --- | --- | --- | --- | --- | --- | --- | --- | --- | --- |
| Index | Sex | | 1.5-2.0 | | | 2.0~2.5 | | | 2.5~ |
| Distribution proportion |  |  | |  | | |  | | |
| Sex | Males | 0.12 | | | 0.11 | | | 0.56 | |
|  | Females | 0.14 | | | 0.13 | | | 0.53 | |
|  | Both | 0.13 | | | 0.12 | | | 0.51 | |
| Regions | Beijing | 0.33 | | | 0.25 | | | 0.21 | |
|  | Tianjin | 0.66 | | | 0.03 | | | 0.00 | |
|  | Gansu | 0.01 | | | 0.00 | | | 0.00 | |
|  | Shanxi | 0.08 | | | 0.07 | | | 0.69 | |
|  | Shaanxi | 0.26 | | | 0.14 | | | 0.12 | |
|  | Jilin | 0.21 | | | 0.16 | | | 0.28 | |
|  | Shandong | 0.16 | | | 0.13 | | | 0.42 | |
|  | Henan | 0.06 | | | 0.06 | | | 0.72 | |
|  | Xinjiang | 0.17 | | | 0.11 | | | 0.17 | |
|  | Shanghai | 0.11 | | | 0.09 | | | 0.50 | |
|  | Jiangsu | 0.12 | | | 0.09 | | | 0.29 | |
|  | Zhejiang | 0.18 | | | 0.13 | | | 0.29 | |
|  | Anhui | 0.36 | | | 0.35 | | | 0.20 | |
|  | Guangdong | 0.16 | | | 0.14 | | | 0.46 | |
|  | Hubei | 0.36 | | | 0.23 | | | 0.15 | |
|  | Jiangxi | 0.03 | | | 0.08 | | | 0.89 | |
|  | Sichuan | 0.06 | | | 0.60 | | | 0.35 | |
|  | Guizhou | 0.10 | | | 0.10 | | | 0.68 | |
|  | Yunnan | 0.13 | | | 0.11 | | | 0.47 | |
| RR_i_**^*^** |  | 1.47 | | | 2.43 | | | 4.00 | |

*The RR_i_ values of different cadmium intervals 1.5~2.0, 2.0~2.5 and 2.5~are 1.47, 2.43 and 4.00, respectively (Filippini et al, 2022).

**Part 5 PAF of cadmium-induced DB in Chinese adolescents and adults**

**Table S3.**  PAF of Cd-induced DM in Chinese adolescents and adults (%)

| Disease | Group | PAF |
| --- | --- | --- |
| Sex | Males | 65.32 |
|  | Females | 64.97 |
|  | Both | 63.60 |
| Regions | Beijing | 53.74 |
|  | Tianjin | 26.44 |
|  | Gansu | 30.78 |
|  | Shanxi | 68.94 |
|  | Shaanxi | 40.74 |
|  | Jilin | 53.87 |
|  | Shandong | 60.21 |
|  | Henan | 69.30 |
|  | Xinjiang | 42.86 |
|  | Shanghai | 62.52 |
|  | Jiangsu | 51.50 |
|  | Zhejiang | 53.71 |
|  | Anhui | 56.13 |
|  | Guangdong | 62.45 |
|  | Hubei | 48.81 |
|  | Jiangxi | 73.67 |
|  | Sichuan | 65.72 |
|  | Guizhou | 68.99 |
|  | Yunnan | 61.96 |

*PAF=Population Attributable Fraction, calculated according to distribution proportion and RR_i_.

**Part 6 Cadmium exposure-related parameters**

| **Table S4.**  Cadmium exposure-related parameters of adolescents and adults in China | | | | | | | | | | | |
| --- | --- | --- | --- | --- | --- | --- | --- | --- | --- | --- | --- |
| Daily intake |  | Smoking | Air  m³/day | Water  L/day | Grains g/d | Vegetable g/d | Potatoes g/d | Fruits  g/d | Meats  g/d | Dairy  g/d | Aquatic  g/d |
| sex | Males | 47.50% | 18.00 | 1.25 | 426.92 | 290.32 | 50.68 | 34.12 | 78.02 | 25.06 | 31.00 |
|  | Females | 2.71% | 14.50 | 1.00 | 367.96 | 267.08 | 45.58 | 32.42 | 57.82 | 23.10 | 26.46 |
|  | Both | 25.66% | 16.10 | 0.80 | 397.44 | 274.33 | 53.61 | 53.37 | 90.52 | 40.69 | 38.31 |
| Regions | Beijing | 27.63% | 16.70 | 0.53 | 361.13 | 325.30 | 42.40 | 105.00 | 104.47 | 113.27 | 26.23 |
|  | Tianjin | 26.90% | 16.70 | 0.61 | 365.40 | 226.20 | 39.90 | 73.90 | 61.60 | 57.95 | 46.40 |
|  | Gansu | 24.27% | 16.20 | 1.10 | 397.03 | 168.40 | 94.97 | 19.20 | 34.13 | 25.13 | 1.03 |
|  | Shanxi | 27.20% | 16.60 | 1.37 | 456.32 | 199.17 | 107.35 | 40.10 | 27.82 | 35.18 | 1.95 |
|  | Shaanxi | 26.10% | 15.60 | 0.79 | 423.89 | 235.70 | 76.03 | 50.70 | 26.44 | 33.44 | 2.29 |
|  | Jilin | 27.37% | 16.60 | 0.32 | 394.40 | 261.00 | 107.05 | 80.30 | 76.05 | 41.40 | 23.60 |
|  | Shandong | 22.87% | 16.50 | 0.68 | 364.33 | 203.91 | 48.73 | 54.30 | 60.14 | 37.41 | 41.00 |
|  | Henan | 23.07% | 16.20 | 1.43 | 478.70 | 286.45 | 26.19 | 45.60 | 40.71 | 20.99 | 11.10 |
|  | Xinjiang | 15.43% | 16.40 | 1.30 | 546.45 | 201.85 | 53.25 | 31.10 | 99.95 | 158.55 | 5.10 |
|  | Shanghai | 22.63% | 16.30 | 0.39 | 337.83 | 314.30 | 22.53 | 109.0 | 132.30 | 101.93 | 132.80 |
|  | Jiangsu | 23.30% | 16.00 | 0.84 | 398.14 | 270.30 | 30.36 | 49.80 | 82.75 | 21.79 | 55.81 |
|  | Zhejiang | 25.20% | 16.00 | 0.51 | 367.61 | 248.33 | 23.33 | 116.00 | 100.68 | 28.80 | 120.70 |
|  | Anhui | 23.87% | 15.90 | 1.31 | 489.25 | 311.60 | 36.85 | 27.70 | 80.35 | 19.70 | 33.70 |
|  | Guangdong | 24.13% | 15.50 | 0.49 | 376.36 | 283.00 | 22.04 | 56.00 | 118.43 | 15.49 | 56.86 |
|  | Hubei | 26.13% | 16.10 | 0.45 | 365.32 | 371.43 | 28.95 | 15.80 | 69.75 | 11.87 | 54.33 |
|  | Jiangxi | 25.30% | 15.60 | 0.40 | 428.63 | 296.97 | 27.60 | 25.80 | 75.83 | 0.73 | 26.27 |
|  | Sichuan | 24.07% | 15.70 | 0.75 | 364.54 | 259.05 | 69.77 | 40.50 | 89.83 | 47.82 | 10.02 |
|  | Guizhou | 33.67% | 15.80 | 0.36 | 404.56 | 271.18 | 48.96 | 16.20 | 80.56 | 10.38 | 8.94 |
|  | Yunnan | 29.67% | 15.80 | 0.50 | 345.82 | 362.60 | 79.72 | 19.30 | 84.86 | 5.18 | 4.98 |
| cadmium contents^*^ |  | 0.009 | 0.37 | 0.039 | 0.037 | 0.024 | 0.004 | 0.122 | 0.004 | 0.205 | 0.009 |

*The smoking rate in China is an average calculated from the results of the National Health Service Survey in 2003, 2008, and 2013(Wang et al., 2019).

^&^ There is an assumption of 15.2 cigarettes per day for smokers and 0.75μg cadmium per cigarette (CDC, 2015; Faroon et al., 2012).

**^#^**The cadmium contents in air, water, grains, vegetables, Potatoes, Fruits, Meats, Dairy and Aquatic

were 0.009μg/m³, 0.37μg/L, 0.039 mg/kg, 0.037mg/kg, 0.024mg/kg, 0.004mg/kg, 0.122mg/kg, 0.004 mg/kg, 0.205mg/kg and 0.009mg/kg, respectively, according to the literature data (Gu, 2019; Luo, 2018 and Liu et al., 2022).

**References**

Filippini T, Wise LA, Vinceti M (2022) Cadmium exposure and risk of diabetes and prediabetes: A systematic review and dose-response meta-analysis. Environ Int, 158, 106920. <https://doi.org/10.1016/j.envint.2021.106920>

Wang M, Luo X, Xu S, Liu W, Ding F, Zhang X, Wang L, Liu J, Hu J, Wang W (2019) Trends in smoking prevalence and implication for chronic diseases in China: serial national cross-sectional surveys from 2003 to 2013. Lancet Respir Med 7(1):35-45. <https://doi.org/10.1016/S2213-2600(18)30432-6>

CDC (2015). 2015 China Adult Tobacco Survey Report. (in Chinese) <https://www.gov.cn/xinwen/2015-12/28/content_5028569.htm>

Faroon O, Ashizawa A, Wright S, Tucker P, Jenkins K, Ingerman L, Rudisill C (2012) Toxicological Profile for Cadmium. Atlanta (GA): Agency for Toxic Substances and Disease Registry (US).

Gu JW (2019) A Review on Heavy Metals in Atmospheric Suspended Particles of China Cities and Its Implication for Future References. Earth and Environment.47(03):385-396. (in Chinese) <https://doi:10.14050/j.cnki.1672-9250.2019.47.045.>

Luo S. Xiang WP (2018) Analysis of metal elements in domestic drinking water. Recyclable Resources and Circular Economy 11(12). 39-41. (in Chinese)

Liu J, Li Y, Li D, Wang Y, Wei S (2022) The burden of coronary heart disease and stroke attributable to dietary cadmium exposure in Chinese adults, 2017. Sci Total Environ 825:153997. <https://doi.org/10.1016/j.scitotenv.2022.153997>
